# Diagnostic Role of Salivary and GCF Nitrite, Nitrate and Nitric Oxide to Distinguish Healthy Periodontium from Gingivitis and Periodontitis

**Published:** 2014

**Authors:** Arash Poorsattar Bejeh-Mir, Hadi Parsian, Maryam Akbari Khoram, Nafiseh Ghasemi, Ali Bijani, Mahmoud Khosravi-Samani

**Affiliations:** 1 Dental Materials Research Center, Dentistry School, Babol University of Medical Sciences, Babol, Iran; 2 Social Determinants of Health Research Center, Babol University of Medical Sciences, Babol, Iran; 3 Biochemistry and Biophysics Department, Babol University of Medical Sciences, Babol, Iran; 4 Private Practice, Mazandaran Province, Iran; 5 Non-Communicable Pediatric Disease Research Center, Babol University of Medical Sciences, Babol, Mazandaran Province, Iran; 6 Dental Materials Research Center, Periodontology and Implantology Department, Babol University of Medical Sciences, Babol, Iran

**Keywords:** Periodontitis, gingivitis, nitric oxide, saliva, gingival crevicular fluid

## Abstract

Diagnosis of subclinical and early stage clinical periodontal dysfunction could prevent from further socioeconomic burden. The aim of this study was to assess the diagnostic applicability of nitric oxide and its end-metabolites in periodontal tissue health and disease. Forty-two patients were enrolled and divided into three groups according to gingivitis (GI) and clinical attachment level (CAL) indices: a healthy group (GI<1, CAL<1), b: gingivitis (GI>1, CAL>1) and c: periodontitis (CAL>1) with 14 patients in each group. Unstimulated saliva and gingival crevicular fluid (GCF) were collected. Samples were evaluated for nitrite, nitrate and total nitric oxide contents with the ELISA method. In addition, CAL, GI, plaque index (PI), decay, missing, filling (DMFT) and bleeding index (BI) scores were also recorded. Except for GCF nitrite content (P= 0.89), there was an increasing trend for measured biomarkers in both saliva and GCF (Periodontitis> gingivitis> healthy periodontium, P< 0.05). Data remained stable after simultaneous adjustment for DMFT and BI scores as confounding factors. Sensitivity, specificity, positive predictive value, negative predictive value, cut point and p- value were as the followings: GCF nitrate (0.71, 0.11, 0.29,0.43, 4.97, P= 0.04), nitric oxide GCF ( 0.64, 0.18, 0.28, 0.5, 10.12, P= 0.04), nitrite saliva (0.93, 0.96,0.93,0.96,123.48, P< 0.001), salivary nitrate (0.93, 0.96, 0.93, 0.96, 123.6, P< 0.001), salivary nitric oxide (0.93, 0.96, 0.93, 0.96, 246.65, P <0.001). Our results revealed that NO plays an important role in the process of destruction of periodontal tissues. Within the limitation of our study, detecting NO biomarker and its end metabolites in saliva is of more value to assess the periodontal health comparing to GCF.

Periodontal disease as one of the most important intraoral diseases causes the loosening of teeth, premature tooth loss and consequently leads to a great socio- economical burden on individuals and community health services levels (1). Of note, increased life expectancy and growing prevalence of systemic diseases with proven adverse effects on periodontium health such as diabetes mellitus are sensed. In addition, greying of Iranian population and reaching the baby boomers of previous decades should be particularly revisited due to the fact that periodontal disease risks and incidence are remarkably and positively correlated with ageing (1-2).

Many inflammatory mediators are known for early and comfortable diagnosis of periodontal disease, such as interleukins, c-reactive protein (CRP), lactate dehydrogenise (LDH) and more recently nitric oxide (NO) (3). Many studies were performed on nitric oxide after discovering its vasodilator effect on rabbit aorta during the last three decades (4). Nitric oxide is produced in the body by two mechanisms: dependent and independent of nitric oxide synthase (NOS) respectively. The independent mechanism of nitric oxide production occurs by entry of nitrite and nitrate contents of foods and their conversion and fermentation by the bacteria of oral cavity and the stomach acid. In the dependent system, three distinct iso-enzymes are involved. Endothelial NOS (i.e, eNOS) is commonly expressed at the cell surface and neuronal NOS (i.e, nNOS) is mainly expressed in brain neurons. These two enzymes are dependent on intracellular calcium and the NO which is produced at pico-molar level disappears in less than 4 seconds. In contrast, inducible NOS (i.e, iNOS) is produced from macrophages that are stimulated by inflammatory cytokines such as IL-1 or IFN γ, TNF-α, that can produce nitric oxide at nano-molar level which is stable for several hours(4).

Several bio-pathologic properties are described for NO such as reducing the inflammatory response; reducing platelet aggregation and vasodilation, bone remodeling and anticariogenic effect. Although NO can act as a double-edged sword, it means that on one side it has anti-inflammatory effects and on the other side acts as a free radical in the form of peroxynitrite which has many tissue damaging effects (4). Salivary nitric oxide can be produced from several sources, including free nerve endings, salivary gland secretory cells, salivary gland endothelial cells and intraoral bacteria (5).

Gingival crevicular fluid (GCF), however, is seemed to be a more reliable source for the identification of periodontal disease. This presumption is based on that it is only affected by periodontal tissues surrounding the teeth comparing to the whole saliva that is secreted from the major salivary glands and therefore composed of GCF at a lower extent. Moreover, whole saliva may be more affected by systemic inflammatory and infectious conditions (6-7). There are some published data that assessed the effect of periodontal inflammation on salivary nitric oxide with somewhat contrasting results (5, 7). Previously, Khosravi et al., introduced a cut-point system to distinguish healthy periodontium from periodontitis based on total salivary nitric oxide content (7). There is no published data about GCF nitric oxide content and its end metabolites (i.e., nitrate and nitrate) in normal periodontium, gingivitis and periodontitis. The aim of the present study was to evaluate diagnostic power of GCF when compared to the whole saliva. Our hypothesis of superiority of GCF over saliva was based on mentioned premise that GCF is directly and solely affected by surroundingperiodontal tissue.

## Methods & Materials

**Study design**

This case-control study was performed on patients who referred to the Department of Periodontology, Babol School of Dentistry during September 2012 to February 2013. Based on previous studies, the number of samples required was 15 patients per group. Considering a 20% loss, a total of 18 patients were included in each group to reach 80% power and a two-tailed type I error of 5% was considered as the statistically significant level. $\left[n=2\left(\mathrm{Z1}-\frac{\alpha }{2}+\mathrm{Z1}-\beta \right)2\frac{\delta^{2}}{d^{2}}=2\left(1.96+0.84\right)=15,N=n\times \frac{120}{100}=18\right]$

**Patient enrollment**

After the periodontal examination of patients admitted to the department, the procedure was described to participants and a written informed consent was obtained. Individuals with a history of smoking, currently taking antibiotics or NSAIDs medications, scaling and root planning in the last 6 months, coagulopathy disorders, salivary gland diseases, use of oral contraceptives or similar hormonal compounds and intraoral neoplasms were excluded.

**Clinical measures**

By recording plaque index (PI, Sillness & Loe (8)), gingival index (GI, Loe & Sillness (9)), bleeding index (BI, Cowel et al. (10)), clinical attachment level (CAL), pocket probing depth (PPD) and DMFT ( decay, missing, filling), the intraoral examination of participated individuals was done. Thereafter, individuals were divided into three groups consisting of healthy (GI<1, CAL<1), gingivitis (GI>1, CAL>1) and periodontitis (CAL>1, with any given GI).

**Sampling and quantitative assessment of NO by ELISA method**

Two milliliters of unstimulated saliva were collected by spitting method as previously reported (7). The participants were asked to avoid eating, drinking, brushing and flossing 90 minutes before collecting the unstimulated salivary samples. Then the individuals poured their saliva into 15 ml test tubes with lids and placed them in sealed flasks with controlled temperature of 0-4^o^C. The flasks were transported to the laboratory immediately, thereafter, they were centrifuged for five minutes (Spectra fuge 24D, Labnet International Inc, Germany) at 3000 rpm. The supernatant was collected and poured into microtubes. Until testing, they were maintained at-80^°^C in the laboratory. GCF collection was performed using paper point # 35. Paper points were inserted into the gingival sulcus until resistance was observed and kept into the sulcus for 30 seconds. Before placing the paper point in the gingival sulcus, the particular teeth were washed and cleaned using cotton rolls and syringes in order to remove dental plaque and saliva. Then they were isolated with cotton rolls. Paper points soaked in blood and saliva, were excluded. Then the paper points were put into microtubes with lids and transferred to the laboratory. They were maintained at -80^°^C until the day of measurement. On the trial day of each mediator, the respected microtubes were removed from -80^°^C temperature and prepared GCF samples were evaluated for NO using enzyme-linked immune sorbent assay (ELISA) method (Acive motif Nitric Oxide Quantitation Kit, North America). In brief, NO is converted to nitrate and nitrite. Nitrate in the sample is converted to nitrite in the presence of nitrate reductase and cofactors. Then, nitrite is assayed using Griess reagent. This two-step assay method provides a simple and sensitive assay for monitoring nitric oxide production.

**Statistics**

Continuous data are presented in mean (±standard deviation). First, the data distribution was tested using the Kolmogorov-Smirnov test. Then, one-way ANOVA, Kruskal-Wallis and univariate general linear model (GLM) tests were used to compare the studied groups with simultaneous adjustment for confounding effects of DMFT, PPD and BI scores (7). Also the predictive power of nitric oxide, nitrate and nitrite of saliva and GCF were assessed by receive operating characteristic (ROC) curve. Diagnostic cut-points were obtained from ROC curve, using the Youden’s index (7). In addition, sensitivity, specificity, positive predictive values (PPV), negative predictive values (NPV), positive likelihood ratio (PLR) and negative likelihood ratio (NLR) were calculated with SPSS (version 19) and Catmaker software.

**Ethical Approval**

The present research was approved by Ethics Committee of Babol University of Medical Sciences and all researches undertook Helsinki treaty.

## Results

**Demographic data**

A total of 42 patients were enrolled and 14 patients were included in each group. Thirteen patients (31 %) were males and 29 patients (69 %) were females. There were 4, 4 and 5 male patients in healthy, gingivitis and periodontitis groups, respectively. In this sense, the groups were not differed significantly by gender (X^2^(2) = 0.22, P = 0.89). The mean age of patients was 38.26 ± 5.50 years ranging from 30 to 50 years. The mean age of healthy, gingivitis and periodontitis were 37.71± 6.16, 38.79± 5.63 and 38.29± 4.50 years, respectively and had no significant difference (F(2, 39)= 0.13, P= 0.88).

**Comparison of DMFT and periodontal indices**

Indices were the highest in periodontitis group and the lowest in normal group. Highest DMFT scores were observed in periodontitis group followed by gingivitis and normal periodontium groups. Data related to the periodontal indices and DMFT are shown in table 1

**Comparison of nitric oxide, nitrate and nitrite in saliva and GCF**

**Fig. 1:**
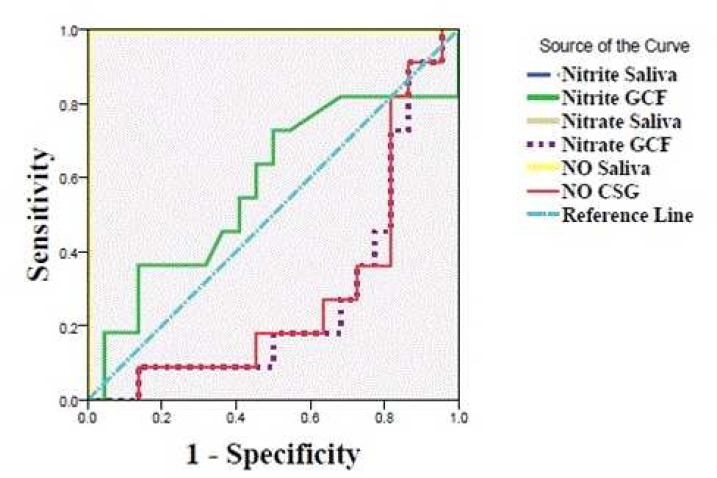
ROC curve to differentiate periodontitis patients from gingivitis patients and healthy individuals. NO: Nitric oxide, GCF: Gingival crevicular fluid.

The average of total amount of nitric oxide and its metabolites in each group is shown in table 2. A significant difference was observed between studied groups, except for nitrite content of GCF which did not reach the statistically significant level. A remarkable decreasing or increasing changing trend was observed for various contents from normal periodontium to gingivitis and periodontitis group, except for the nitrite content of GCF that did not reveal such a constant pattern. Also the comparison of data between the groups remained stable after the adjustment of nitrate and nitrite and NO of saliva and GCF by DMFT, BI and nitrate and nitrite and NO of GCF by PPD amount, the BI scores of teeth from which samples were collected (salivary nitrate: P=0.007, salivary nitrite: P<0.001, salivary NO: P=0.007, GCF nitrate: GCF nitrite: P=0.15 and GCF NO: P<0.001). Predictive power of nitric oxide and its metabolites in GCF and saliva for detection of the periodontitis is shown in Figure 1 and Table 3.

**Table 1:** Mean (± Standard Error) of Clinical Parameters in the Studied Groups

**Statistical significance**	**Periodontitis** **Group** ** (n=14)**	**Gingivitis** **Group** **(n=14)**	**Healt** **y(n=14) ** **Group**	**Index**
X^2^(2)=2.04, P<0.001†	2.21(±0.073)	0.559(±0.073)	0.427(±0.092)	CAL(mm)
F(2,39)=16.09, P<0.001 B††	2.02(±0.146)	1.44(±0.067)	1.32(±0.016)	PPD(mm)
F(2,39)=20.25, P<0.001††	1.08(±0.21)	0.923(±0.052)	0.023(±0.007)	BI
F(2,39)=75.53 P<0.001††	0.937±(0.13)	1.44(±0.06)	0	GI
F(2,39)=20.83 P<0.001††	1.09±(0.15)	1.01(±0.05)	0.2460(±0.077)	PI
F(2,39)=38.22 P<0.001††	17.07(±0.78)	11.85(±0.75)	7.14(±0.86)	DMFT

† Kruskal –Wallis H test ,

†† One-way ANOVA

**Table 2:** Mean (± SE) of ​​Measured Mediators in GCF and Saliva of Assessed Groups

**Statistical significance**	**Periodontitis group ** ***(n=14)***	**Gingivitis group** ***(n=14)***	**Healthy group** *** (n=14)***	
F(2,36)=0.53, P=0.867	11.54(±2.82)	9.64(±2.02)	10.69(±2.62)	GCF Nitrite
F(2,39)=4.26, p=0.015	5.39(±2.13)	10.45(±2.10)	15.92(±2.97)	GCF Nitrate
F(2,39)=4.94 P=0.019	12.16(±3.88)	20.36(±4.30)	32.05(±5.98)	GCF NO
F(2,38)=117.69 P<0.001	173.26(±9.26)	79.64(±4.62)	33.19(±4.69)	Salivary nitrite
F(2,39)=29.05 P<0.001	172.99(±9.26)	79.36(±4.62)	50.39(±17.79)	Salivary nitrate
F(2,39)=29.66 P<0.001	346.25(±18.52)	159(±9.25)	100.95(±35.60)	Salivary NO

**Table 3:** Predictive Power of Nitric Oxide and its Metabolites in GCF and Saliva.

**NLR** ‡‡	**PLR** ‡	††† **NPV**	†† **PPV**	**Specifity**	**Sensitivity**	**Statistical** ** significance**	**AUC** †	
						P=0.49	0.57	GCF nitrite
2.67	0.8	0.43	0.29	0.11	0.71	P=0.04	0.27	GCF nitrate^a^
2	0.78	0.50	0.28	0.18	0.64	P=0.04	0.28	GCF NO^b^
0.07	26	0.96	0.93	0.96	0.93	P<0.001	1	Salivary nitrite^c^
0.07	26	0.96	0.93	0.96	0.93	P<0.001	1	Salivary nitrate^d^
0.07	26	0.96	0.93	0.96	0.93	P<0.001	1	Salivary NO^e^

a-e represent cutpoint values:

† : area under curve,

†† : positive predictive value,

††† :negative predictive value,

‡ : positive likelihood ratio,

‡‡ negative likelihood ratio.

a :4.97,

b : 10.12,

c :12348,

d :123.6,

e :246.65

## Discussion

In this study, the diagnostic value of nitric oxide (NO) in saliva and GCF were examined to assess the periodontal health status. Results showed that the overall levels of nitric oxide, nitrite and nitrate were higher in saliva compared to GCF. Also, the salivary nitric oxide increased in the order of healthy subjects, gingivitis patients and those suffering from periodontitis while GCF nitric oxide decreased in the same order.

To our knowledge, NO and its metabolites in saliva and GCF are being evaluated and compared among the three groups of healthy subjects, gingivitis and periodontitis patients for the first time. Unstimulated salivary samples were collected as the mastication affects it (11). The levels of NO and its metabolites were significantly different among three groups, except for GCF nitrite levels (P= 0.59), that even after adjustment with BI, differences did not reach a significant level (P = 0.15). All the above salivary levels were adjusted by DMFT index, BI and the GCF levels were adjusted by BI, PPD.  It has been suggested that NO reflects the dynamic state of the patient to a static state. Thus, the pattern of disease progression in gingivitis patients with high BI (active) is different from that in periodontitis patients with high CAL and low BI (dormant advanced periodontitis). So all levels were moderated with BI as a key indicator of disease activity and affecting factor on the amount of NO and its metabolites. (11-13). Due to previously documented relationships between DMFT and the salivary NO levels as an antibacterial agent, the groups were moderated with the mean DMFT of their respective patients (5, 14-15). Similarly, the reason for adjustment for PPD scores was that several researchers proved the relationship between NO and PPD (7, 16-17). 

The pathophysiological role of NO in periodontal disease was introduced in 2000 by Ozmeric et al. (18). It has been shown that bacterial lipopolysaccharide in the wall of periopathogenic bacteria causes death and apoptosis of periodontal ligament (PDL) through increased iNOS and phosphorylation of C-Jun N-terminal kinase (19-20). By these means, it may confer a localized mircovasculopathy, lasting ischemia and consequent permanent damage to endothelial and surrounding periodontal tissues (4). The amount of salivary nitrate, nitrite and NO in periodontitis patients was higher than that in gingivitis patients and healthy individuals which was similar to the results reported by Reher et al, Parwani et al. and Menaka et al. (16-17, 21). However, contrary findings were reported by Artese et al. and Ozer et al. (22-23). It seems that these differences are due to the lack of moderation with BI as an effective factor and also DMFT in patients of various groups. Inconsistencies among evaluated groups in various studies could also explain part of the differences.

GCF NO was reduced in order in the three studied groups and the GCF nitrate level was the highest in gingivitis group and the lowest in the periodontitis group. Ozer et al., in their study reported the greatest amount of NO in gingivitis patients (23). So according to their opinion, probably the secreted substances in periodontitis suppress the production of NO (23). Also, as a defence molecule, it may be consumed when disease progresses to combat the interfering oxidative and infectious process (3, 24). This could be due to the fact that consumption of NO as an antibacterial agent is for controlling the bacteria that reduce its level (3).

In our research, the amount of total NO, nitrite and nitrate of saliva were found to be more sensitive biomarkers than GCF content of total NO, nitrite and nitrate, rejecting the hypothesis of the current project. First it was thought that GCF may possess higher capacity to reflect the periodontal diseases, as its biomarkers are mainly secreted from the surrounding individual periodontal tissues. In contrast, saliva is affected from various tissues including periodontal tissue, caries, systemic diseases and salivary glands. We presume that low secreted amount of aforementioned biomarkers into crevicular sulcus may contribute to explain our findings. In addition, the reason for lack of difference in GCF nitrite may be that nitrite is more reflective of eNOS while nitrate is more associated with iNOS (3, 25). ROC curves showed that the saliva is more reliable in differentiation of periodontitis from gingivitis and healthy periodontium. The amount of salivary NO with cut point= 246.65 with respective sensitivity, specifity, PPV and NPV of 0.93, 0.96, 0.93 and 0.96 can differentiate periodontitis patients from gingivitis patients and healthy individuals. It is higher than the balanced cut point of 101 introduced by Khosravi Samani et al., with respective sensitivity, specificity, PPV and NPV of 0.7, 0.96, 0.66 and 0.76. In their study, they used conventional Griess reaction and not the ELISA method. Also, they included normal individuals and periodontitis patients, but no gingivitis group was enrolled (7).

The present investigation is limited in some aspects. Patients with advanced periodontitis were not included. A better cut point could be presented with higher sample size and including patients with CAL more than 5 mm. Another issue in this particular group is that as previously shown there is an exponential and not linear correlation with PPD and NO content. This fact may be related to more remarkable influence over the GCF ingredients of advanced periodontitis group with deeper PPD (7). Our final number of enrolled patients was lesser than the primary design that had lessen the power of our analysis. As previously suggested, simultaneous measuring of NOS substrates such as available oxygen, arginine and reactive oxygen species (ROS) could reflect the competency of NOS systems more accurately (12). This could be accomplished by developing laboratory systems that could measure eNOS and iNOS within saliva and GCF eliminating the need for tissue sampling.

In conclusion, nitric oxide plays a role in the destruction of periodontal tissues. By considering the limitations of current study, the measured levels in the patients saliva compared with their GCF are of higher paraclinical and diagnostic value. Nevertheless, more sensitive techniques with measurement of iso-enzymes of nitric oxide synthase may warrant more conclusive assumptions.
